# Prioritizing chronic obstructive pulmonary disease (COPD) candidate genes in COPD-related networks

**DOI:** 10.18632/oncotarget.21874

**Published:** 2017-10-17

**Authors:** Yihua Zhang, Wan Li, Yuyan Feng, Shanshan Guo, Xilei Zhao, Yahui Wang, Yuehan He, Weiming He, Lina Chen

**Affiliations:** ^1^ College of Bioinformatics Science and Technology, Harbin Medical University, Harbin, Heilongjiang Province, China; ^2^ Institute of Opto-Electronics, Harbin Institute of Technology, Harbin, Heilongjiang Province, China

**Keywords:** chronic obstructive pulmonary disease, gene prioritization, metabolic network, protein-protein interaction network, functional information

## Abstract

Chronic obstructive pulmonary disease (COPD) is a multi-factor disease, which could be caused by many factors, including disturbances of metabolism and protein-protein interactions (PPIs). In this paper, a weighted COPD-related metabolic network and a weighted COPD-related PPI network were constructed base on COPD disease genes and functional information. Candidate genes in these weighted COPD-related networks were prioritized by making use of a gene prioritization method, respectively. Literature review and functional enrichment analysis of the top 100 genes in these two networks suggested the correlation of COPD and these genes. The performance of our gene prioritization method was superior to that of ToppGene and ToppNet for genes from the COPD-related metabolic network or the COPD-related PPI network after assessing using leave-one-out cross-validation, literature validation and functional enrichment analysis. The top-ranked genes prioritized from COPD-related metabolic and PPI networks could promote the better understanding about the molecular mechanism of this disease from different perspectives. The top 100 genes in COPD-related metabolic network or COPD-related PPI network might be potential markers for the diagnosis and treatment of COPD.

## INTRODUCTION

Chronic obstructive pulmonary disease (COPD) is the third leading cause of morbidity and mortality worldwide [1]. As a complex disease, COPD is caused by many factors, including smoking, advanced age, systemic inflammation, and especially disturbances of metabolism [2] and protein-protein interactions (PPIs). For example, glucose metabolism disturbances were more observed in COPD patients than in control individuals [3]. An elevated energy metabolism was also detected in COPD patients [4]. In the COPD pathogenesis, the interaction between CCR6 and its ligand CCL20 promotes the effect of dendritic cells [5]. The interaction of TPP1 with the Sirtuin 1 complex could be disrupted by cigarette smoke. This caused reduced level of TPP1 on telomeres in lungs from COPD patients [6].

Molecular changes occurring in the process of complex diseases could be represented in terms of metabolic networks [7] and PPI networks, which have been used in many researches from various aspects. Shang et al. identified disease-related metabolites from a global metabolic network based on the assumption that the metabolites related to the same disease tend to be modularized in metabolic networks. Good performance and robustness were achieved for different disease classes, especially for respiratory diseases [8]. By integrating coexpression networks with metabolic networks, Ni et al. developed a computational method to predicted key enzyme-coding genes in both Parkinson’s disease and Huntington’s disease. These predicted metabolic genes might act as novel biomarkers the diagnosis and potential therapeutic treatments of these diseases [9]. Wang et al. identified 23 novel genes potentially related to infertility from a human PPI network based on previously validated infertility-related genes. The identified genes were strongly related to dysfunction of four main biological processes of fertility [10]. By integrating the gene expression profile data and PPI data, Huo et al. constructed two coexpression PPI networks in a coronary heart disease (CHD) state and a non-CHD state. They found that the treatment of CHD with Danshensu may be partly attributed to the regulation of immunization and blood circulation. Several potential therapeutic targets for CHD were also identified [11]. Integrating other information into networks could help to better reveal disease mechanisms. Blais et al. predicted biomarker changes in response to drugs by integrating transcriptomics data to metabolic networks for hepatocytes. Their results were validated with literature-based evidence and new experimental data [12]. Zeng et al. used a novel relevance measure to prioritize candidate disease genes based on a heterogeneous network integrating PPI and phenotype information. The 3-fold experiments showed that their methods were better than or similar to existing methods [13]. Transcriptome data were integrated to PPI networks of differentially expressed genes in peripheral blood mononuclear cells and pancreatic β-cellsto to identify key genes associated with Type 1 diabetes risk [14]. Many researches have found that genes with similar functions are more likely to be associated with similar diseases [15–17]. Therefore, it is necessary to further integrate functional information into disease-related networks to study the mechanism of diseases.

In this paper, two weighted COPD-related networks were constructed base on COPD disease genes and functional information. Candidate genes in each COPD-related network were prioritized by making use of a gene prioritization method, respectively. The top-ranked genes in the COPD-related metabolic network or COPD-related PPI network could reflect the molecular mechanism of COPD and might be potential markers for its diagnosis and treatment.

## RESULTS

Base on COPD disease genes, a COPD-related metabolic network and a COPD-related PPI network were constructed, respectively. Nodes and edges of these two COPD-related networks were weighted by integrating functional information. For genes in each COPD-related network, disease risk scores were calculated taking the transfer of disease risks into consideration.

### Gene prioritization

COPD candidate genes were prioritized in each COPD-related network according to their risk scores in descending order (details in Methods). The top-ranked genes in each network had higher disease risk scores and were more associated with COPD. To further demonstrate the relationships between these genes and COPD, literature validation and functional enrichment analysis were applied for the top 100 genes in each COPD-related network.

For the top 100 genes in the COPD-related metabolic network, it was found that higher ranked genes were validated with higher proportion in literature. That is, 56% of the top 100 genes, 66% of the top 50 genes and 90% of the top 10 genes were associated with COPD in literature, such as CYP2E1 (Rank: 1), CYP2C9 (Rank: 4), NOS1 (Rank: 5) and CYP1B1 (Rank: 8). These associations have been explained in our previous work [18].

For top genes in the COPD-related PPI network, 61% of the top 100 genes and72% of the top 50 genes have been validated by literature, though only 40% of the top 10 genes were validated to be associated with COPD by literature. COPD was independently associated with lower prevalences of EGFR (Rank: 2) mutations [19]. Human COPD lungs had decreased protein levels of CTNNB1 (Rank: 4), which was positively correlated with pulmonary function [20, 21]. The protein level of UBB (Rank: 7) was significantly different between control and COPD lung tissue by western analysis [22]. Higher SRC (Rank: 8) activation was measured in small airway epithelial cells from patients with COPD compared with healthy control subjects, which indicated that the activation of SRC promotes COPD-related processes [23].

**Figure 1:**
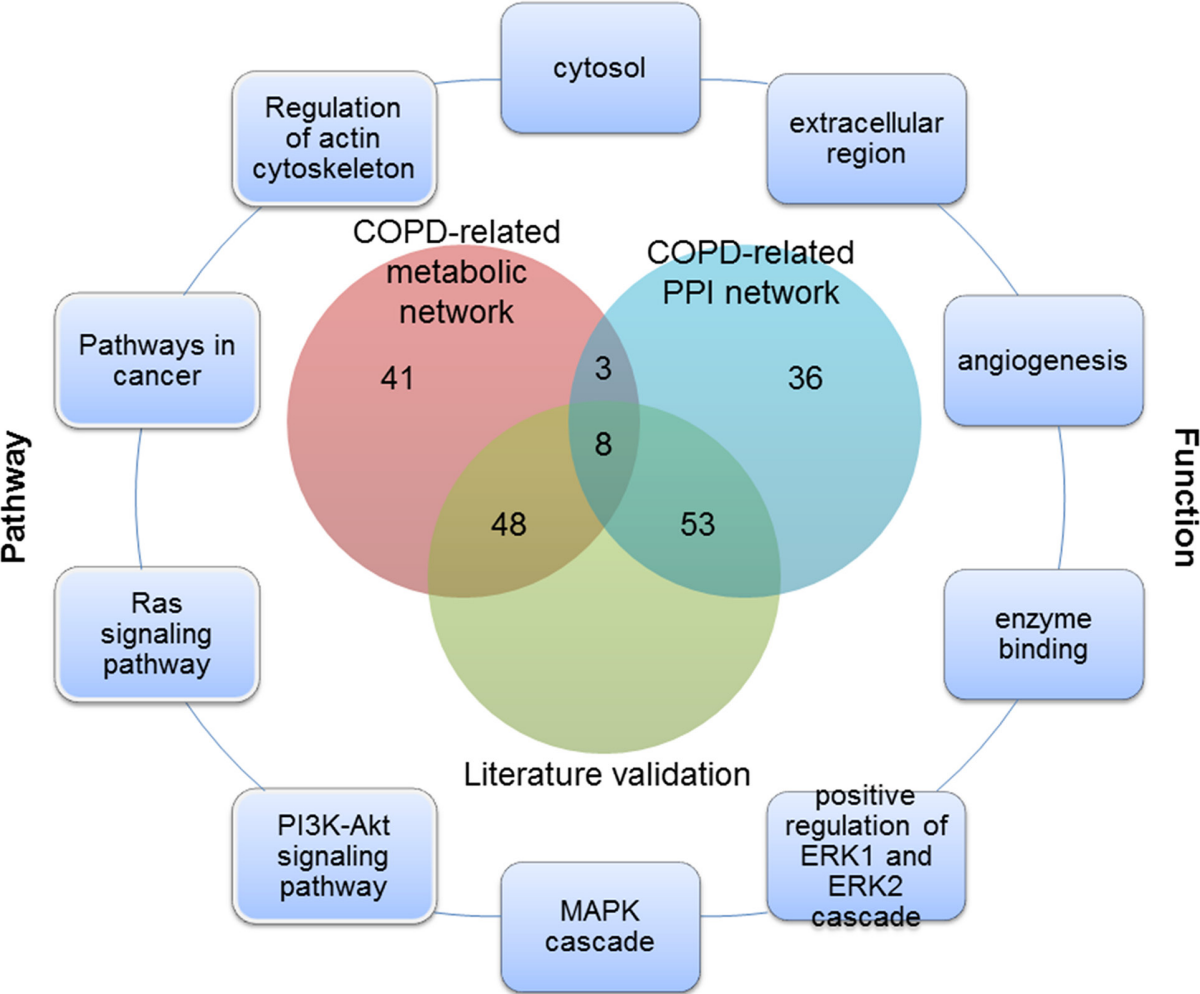
The overlap of the top 100 genes and literature validation from the COPD-related metabolic and PPI networks, and part of COPD-related functions and pathways for these 11 common genes

Of the top 100 genes in two COPD-related networks, 11 genes were common (Figure 1), 8 of which have been validated by literature. For example, SOD1 was supposed to participate in the antioxidant defense of lungs in COPD patients, since its protein levels were found to be significantly higher in COPD patients than in those with no COPD [24]. Quantitative digital image analysis revealed increased cytoplasmic expression of FGF2 in bronchial epithelium and airway smooth muscle in COPD patients compared with controls [25]. Zanini et al. also found that FGF2 were significantly increased in COPD patients as compared to controls [26]. These common genes played important roles in COPD. It was speculated that unique genes of the top 100 genes prioritized from COPD-related metabolic and PPI networks could reflect the molecular mechanism of COPD from different perspectives. These common or unique genes could be involved in various COPD-related processes about metabolism or protein interactions.

**Figure 2:**
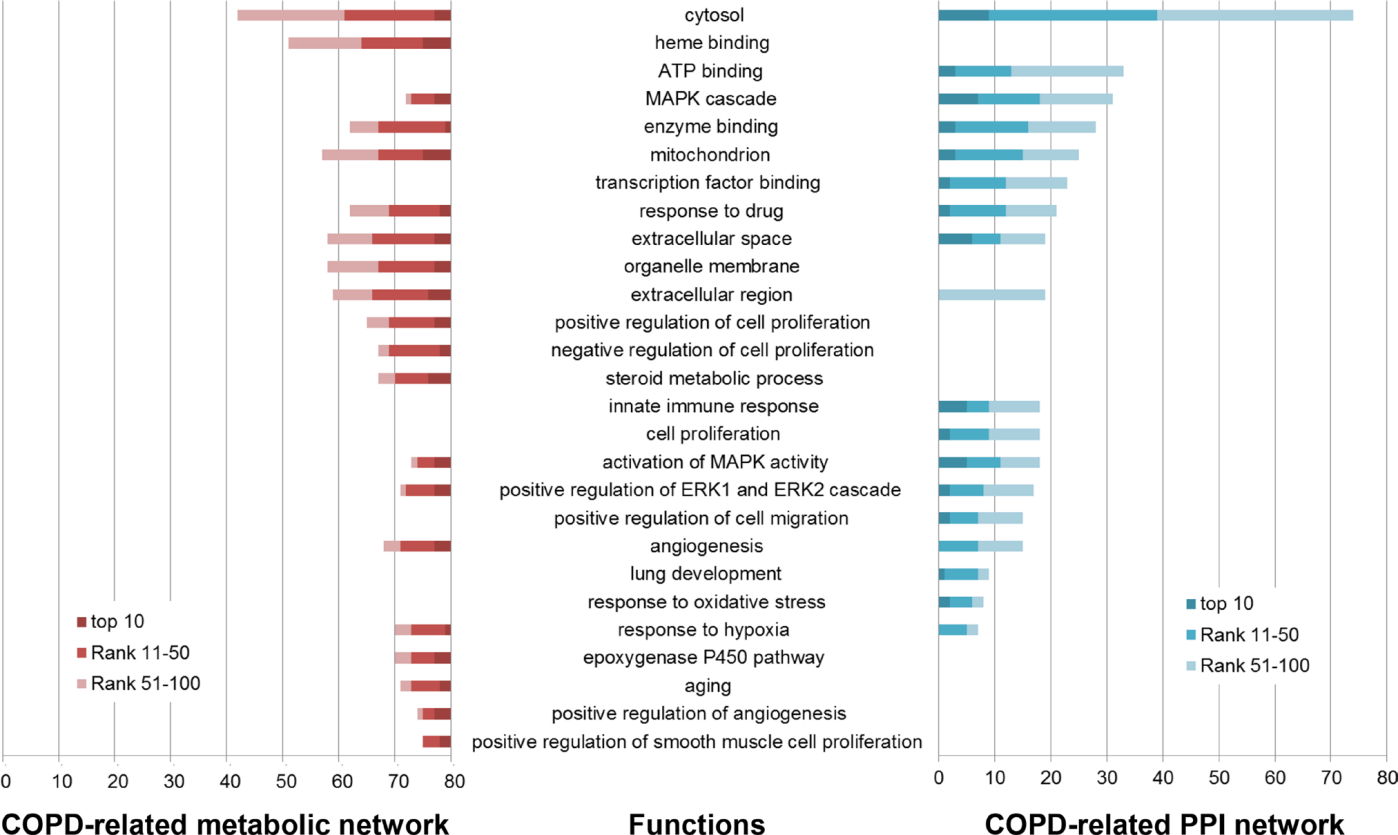
Some of COPD-related GO functions significantly enriched by the top 100 genes in the COPD-related metabolic network (left) and the top 100 genes in the COPD-related PPI network (right) GO functions (horizontal axis) were significantly enriched by the top 100 genes (the number in the vertical axis) using DAVID (Benjamini corrected *P* value < 0.05).

Functions annotated by COPD disease genes were defined as COPD-related functions. 45 COPD-related functions were significantly enriched by the top 100 genes in the COPD-related metabolic network and the top 100 genes in the COPD-related PPI network (Benjamini adjusted *P* value < 0.05) (some are illustrated in Figure 1 and Figure 2), including “Angiogenesis” and “extracellular regions”. The associations of these functions and COPD have been explained in our previous work [18].

153 GO functions were significantly enriched by the top 100 genes in the COPD-related metabolic network (Benjamini adjusted *P* value < 0.05), 86 (56.209%) of which were COPD-related (some are illustrated in Figure 2). “Heme binding” was one of these COPD-related functions, whose role in COPD has been described in our previous work [18]. The “organelle membrane”-permeant iron chelator deferiprone could contribute to alleviate experimental COPD [27]. The “steroid metabolic process” was involved by downregulated genes screened from a dataset including three COPD samples and three normal samples [28].

The top 100 genes in the COPD-related PPI network were significantly enriched in 541 GO functions (Benjamini adjusted *P* value < 0.05). 223 (41.220%) were COPD-related functions (some are illustrated in Figure 2). “Transcription factor binding” site (TFBS) analysis confirmed that multiple COPD eQTL SNPs disrupted TFBS [29]. Dampening the “innate immune response” to smoking played a critical role in modifying pulmonary inflammation and lung remodeling, which might slow the progression of COPD [30]. Nasal epithelial cells are involved in many airway diseases, including asthma and COPD, through their “innate immune response” and interaction with immune and airway stromal cells [31]. Bush et al. found that genes important in “lung development” and early wheezing were implicated in COPD [32].

**Figure 3:**
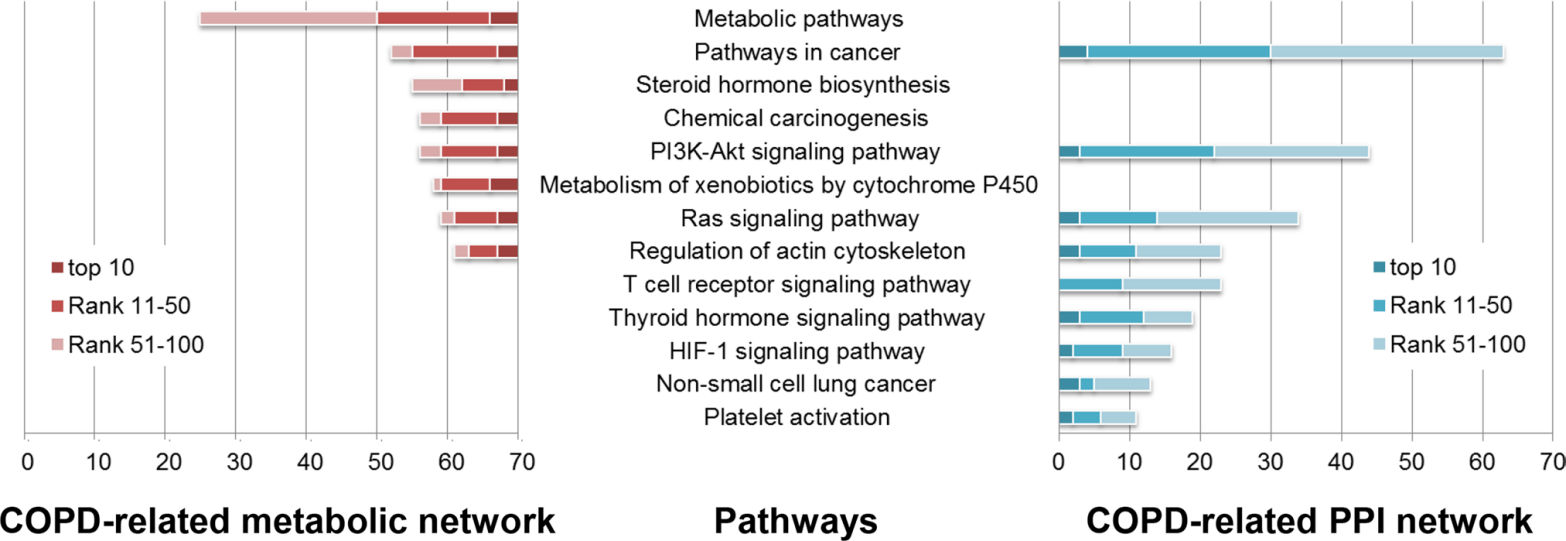
Some of COPD-related KEGG pathways significantly enriched by the top 100 genes in the COPD-related metabolic network (left) and the top 100 genes in the COPD-related PPI network (right) KEGG pathways (horizontal axis) were significantly enriched by the top 100 genes (the number in the vertical axis) using DAVID (Benjamini corrected *P* value < 0.05).

In COPD-related pathways that were annotated by COPD disease genes, 18 could be significantly enriched by the top 100 genes in the COPD-related metabolic network and the top 100 genes in the COPD-related PPI network (Benjamini adjusted *P* value < 0.05) (Some are illustrated in Figures 1 and 3). These pathways included both metabolic pathways and signaling pathways, which indicated their involvement in COPD. Through “regulation of the actin cytoskeleton” and up-regulation of integrin-β1, normal contractile function could be restored for COPD patients [33]. The “PI3K/Akt signaling pathway” was required for epithelial-mesenchymal transition in small airway fibrosis of COPD patients [34].

The top 100 genes in the COPD-related metabolic network were significantly enriched in 34 KEGG pathways (Benjamini adjusted *P* value < 0.05), 32 (94.118%) of which were COPD-related (Some are illustrated in Figure 3). Most of these pathways were “metabolic pathways”, such as “Steroid hormone biosynthesis”, “Metabolism of xenobiotics by Cytochrome P450” and “retinol metabolism”. The associations of these pathways and COPD have been explained in our previous work [18].

104 KEGG pathways were significantly enriched by the top 100 genes in the COPD-related PPI network (Benjamini adjusted *P* value < 0.05). 78 (75%) were COPD-related pathways (Some are illustrated in Figure 3), most of which were signaling pathways. “T cell receptor signaling” molecules were down-regulated in COPD pulmonary CD8 cells [35]. In the development of “Non-small cell lung cancer”, COPD and smoking played a vital role. Local progression and metastasis of “Non-small cell lung cancer” has been associated with the epithelial mesenchymal transition, which was implicated in COPD pathogenesis [36]. “Platelet activation” was a potential therapeutic target in patients with COPD aiming to reduce their risk of thrombosis or other cardiovascular events [37–39].

These results demonstrated the top-ranked genes in each COPD-related network were more associated with COPD, and could be enriched in COPD-related functions or pathways.

### Performance evaluation and comparison

**Figure 4:**
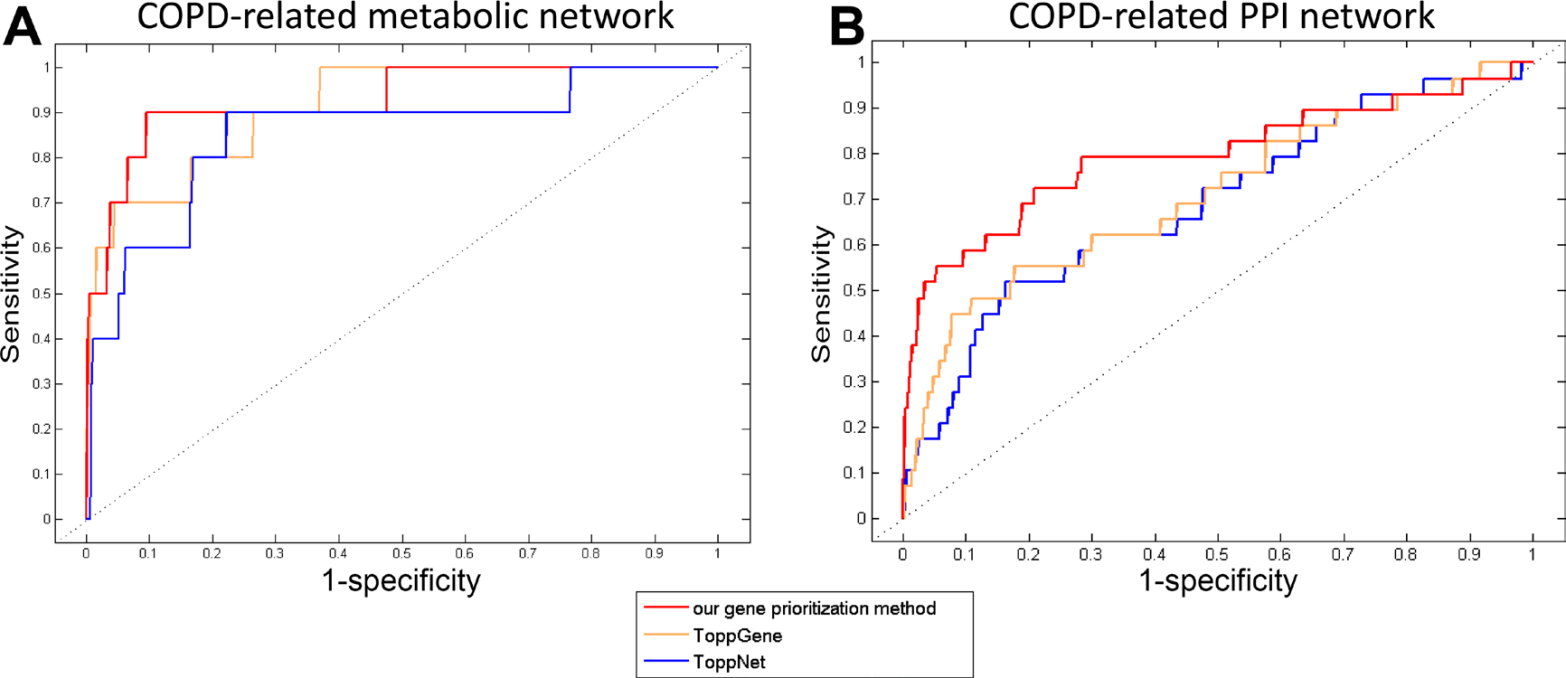
The ROC curves of our gene prioritization method, ToppGene and ToppNet for COPD-related (**A**) metabolic and (**B**) PPI networks.

The performance of our gene prioritization method was assessed for each COPD-related network using leave-one-out cross-validation (LOOCV) (details in Methods). Then, our method was compared with ToppGene and ToppNet based on the area under the receiver operating characteristic (ROC) curve (AUC). ToppGene and ToppNet are two tools in the ToppGene Suite (https://ToppGene.cchmc.org) [40] for prioritizing candidate genes based on a set of disease genes considering various factors, such as GO annotations and protein interactions. It was showed that AUCs of our gene prioritization method for both COPD-related networks (0.949 and 0.799) were higher than those of ToppGene (0.912 and 0.714) and ToppNet (0.854 and 0.687) (Figure 4).

The three methods were also compared on the validated proportion of their top 100 genes in literature. For genes from the COPD-related metabolic network, the proportions for ToppGene and ToppNet have been described in our previous work (Supplementary Figure 1) [18], which were less than that for our gene prioritization method. For genes from the COPD-related PPI network, 47% of the top 100, 42% of the top 50, and 50% of the top 10 genes prioritized by ToppGene were validated, while 31% of the top 100, 30% of the top 50, and 30% of the top 10 genes prioritized by ToppNet were validated to be involved in COPD (Supplementary Figure 2). Most of these proportions were less than those of our gene prioritization method (61%, 72% and 40%).

The performance of the three methods were further compared on enriched COPD-related function or pathway proportion of the top 100 genes employing functional enrichment analysis (Supplementary Table 1). For genes from the COPD-related metabolic network, the comparison of the numbers and proportions of COPD-related functions or pathways have been described in our previous work [18]. For genes from the COPD-related PPI network, the top 100 genes of our gene prioritization method could be enriched in more COPD-related functions or pathways than those of ToppGene and ToppNet, although the proportions were slightly smaller.

These results showed that the top-ranked genes of our gene prioritization method had better performance on AUC of LOOCV, literature validation and COPD-related function or pathway proportion. Thus, these genes were more associated with COPD than those of ToppGene and ToppNet for both COPD-related networks.

## DISCUSSION

In this paper, COPD candidate genes were prioritized in two weighted COPD-related networks according to their risk scores by using a gene prioritization method, respectively. Literature validation and functional enrichment analysis were assessed for the top 100 genes from each COPD-related network. The performance of the gene prioritization method was superior to that of ToppGene and ToppNet on AUC of LOOCV, literature validation and COPD-related function or pathway proportion for their top 100 genes.

To further exhibit the classification performance of the top-ranked genes in two COPD-related networks, a support vector machine with linear kernel was employed to classify samples of a COPD-related expression profile GSE57148. The profile was obtained from Gene Expression Omnibus (GEO, https://www.ncbi.nlm.nih.gov/geo/) [41], which contained 98 COPD patients and 91 normal controls. The classification process was conducted for the top 10 (the same number as COPD disease genes in the COPD-related metabolic network), the top 29 (the same number as all COPD disease genes) and the top 100 genes in two COPD-related networks, respectively. Then the same classification process was conducted for 10 COPD disease genes in the COPD-related metabolic network and 29 COPD disease genes in the COPD-related PPI network (see Data). AUC was used to compare their classification performance (Table 1). It was showed that the classification performance of the top 10 genes in both COPD-related networks was better than that of 10 COPD disease genes. The classification performance of 29 COPD disease genes was better than that of the top 29 genes in the COPD-related metabolic network, while that of the top 29 genes in the COPD-related PPI network was even better. The top 100 genes in two COPD-related networks could both classify samples with good performance.

**Table 1:** The classification performance (AUC) of top 10, 29 and 100 genes in both COPD-related networks, and of 10 and 29 COPD disease genes

	10^a^	29^a^	100^a^
COPD- related metabolic network	0.729	0.810	0.789
COPD- related PPI network	0.853	0.896	0.932
COPD disease genes	0.725	0.837	−

^a^ The number of top-ranked genes used to classify samples.

The performance of the top 100 genes in the COPD-related metabolic network was better on the numbers and proportions of enriched functions or pathways and AUC of LOOCV than those from the COPD-related PPI network, while the performance of the top 100 genes in the COPD-related PPI network was better on literature validation and the classification performance than those from the COPD-related metabolic network. These results indicated that the top-ranked genes prioritized from these two COPD-related networks could reflect the molecular mechanism of COPD from different perspectives by participating in various COPD-related processes about metabolism or protein interactions.

To conclude, COPD candidate genes were prioritized in COPD-related networks using the gene prioritization method. The correlation of the top 100 genes and COPD was validated by literature and functional enrichment analysis. Compared with ToppGene and ToppNet, our gene prioritization method had better performance. The top-ranked genes prioritized from COPD-related metabolic and PPI networks could promote the better understanding about the molecular mechanism of this disease from different perspectives. The top 100 genes in either COPD-related network might be potential markers for the diagnosis and treatment of COPD.

## MATERIALS AND METHODS

### Data

COPD disease genes were obtained from databases and literature, including Online Mendelian Inheritance in Man (OMIM, https://www.omim.org/) [42], the Disease Ontology (DO, http://disease-ontology.org/) [43], Phenotype-Genotype Integrator (PheGenI) (https://www.ncbi.nlm.nih.gov/gap/phegeni) [44], DISEASES (http://diseases.jensenlab.org/) [45] and Menche’s research [46]. A total of 29 COPD disease genes were collected for further analysis.

Gene functional information was extracted as all annotation terms for human genes in three ontologies, i.e. biological processes, molecular functions and cellular components, from Gene Ontology (GO, http://www.geneontology.org/) [47].

### Construction of weighted COPD-related networks

Based on these COPD disease genes, two weighted COPD-related networks were constructed. One was a COPD-related metabolic network, which was built using COPD disease genes and their direct interactors extracted from an integrated human metabolic network as described in our previous work [18]. The COPD-related metabolic network contained 1361 genes and their 6601 interactions, 10 of which were COPD disease genes, and others were candidate genes. The other was a COPD-related PPI network, which was retrieved using COPD disease gene products and their interacting partners from the STRING database (http://string-db.org/) [48]. The COPD-related PPI network was comprised of 7791 interactions between 3740 proteins (gene products). All of 29 COPD disease genes were in the network, and other genes were candidate ones.

Weights for genes and interactions (nodes and edges of these COPD-related networks) were calculated by integrating functional information as in our previous work [18].

### Prioritization of candidate genes

To prioritize candidate genes in each COPD-related network, disease risk score of each gene was obtained taking the transfer of disease risks into consideration, respectively:

$$D^{\left(i=1\right)}=\left(1-\beta \right)QD^{\left(i\right)}+\beta D^{\left(0\right)}$$

where *D(i)* is the vector of risk scores of all genes at step *i*, and β∈(0,1) is a parameter to measure the importance between genes and interactions. After assessing the performance using *β* = 0.1, 0.2,⋯, 0.9, *β* =0.1 was chosen as the optimal parameter. *Q* is the disease risk transition probability matrix, whose element q(g|h), the disease risk going from gene *h* to gene *g* , was defined as

$$q\left(g|h\right)=\frac{w_{\left(h,g\right)}}{\sum_{l\in neighbor\left(h\right)}w_{\left(l,g\right)}}$$

where *w*_(h,g)_ is the interaction weight between interacting genes *h* and *g* , and *neighbou(h)* is the set of genes that interact with gene *h*. *D*^(0)^ is the vector of initial disease risk scores for all genes. Each element of *D*^(0)^, i.e. score d^g(0)^ for gene *g* in each COPD-related network, was calculated as follows:

$$d_{g}^{\left(0\right)}=\frac{w_{g}}{\sum_{m\in \text{COPD}-\text{related metabolic}/\text{PPI network}}w_{m}}$$

The process was carried out until the difference between *D*^(i)^ and *D*^(i+1)^ was less than a threshold, 10^–9^. Candidate genes from each COPD-related network were prioritized according to their risk scores in descending order.

To examine the association between the top-ranked genes and COPD, literature validation was performed for the top 100 genes in each COPD-related network in literature of PubMed (http://www.ncbi.nlm.nih.gov/pubmed). Then, functional enrichment analysis was applied for the top 100 genes using the Functional Annotation Tool in the Database for Annotation, Visualization and Integrated Discovery (DAVID, http://david.abcc.ncifcrf.gov/) v6.8 [49, 50]. GO functions and KEGG pathways with adjusted *P* value (Benjamini) less than 0.05 were considered significant.

### Evaluation and comparison of the performance

LOOCV was carried out to assess the performance of our gene prioritization method as described our previous work [18]. The ROC curves were plotted and AUC was computed based on the ranks of test genes. These results were compared with those of ToppGene and ToppNet using genes from COPD-related metabolic and PPI networks, respectively.

To compare with the top 100 genes of our gene prioritization method, literature validation and functional enrichment analysis for the top 100 genes prioritized by ToppGene and ToppNet were also performed.

## SUPPLEMENTARY MATERIALS FIGURES AND TABLE


