# Supplementary material for: Prioritizing chronic obstructive pulmonary disease (COPD) candidate genes in COPD-related networks
Source: Oncotarget. 2017 Oct 17;8(61):103375–84. doi: 10.18632/oncotarget.21874 (PMC5732734; doi:10.18632/oncotarget.21874)
Supplement: Supplementary file 1 [file oncotarget-08-103375-s001.pdf]

Prioritizing chronic obstructive pulmonary disease (COPD) candidate genes in COPD-related networks

SUPPLEMENTARY MATERIALS

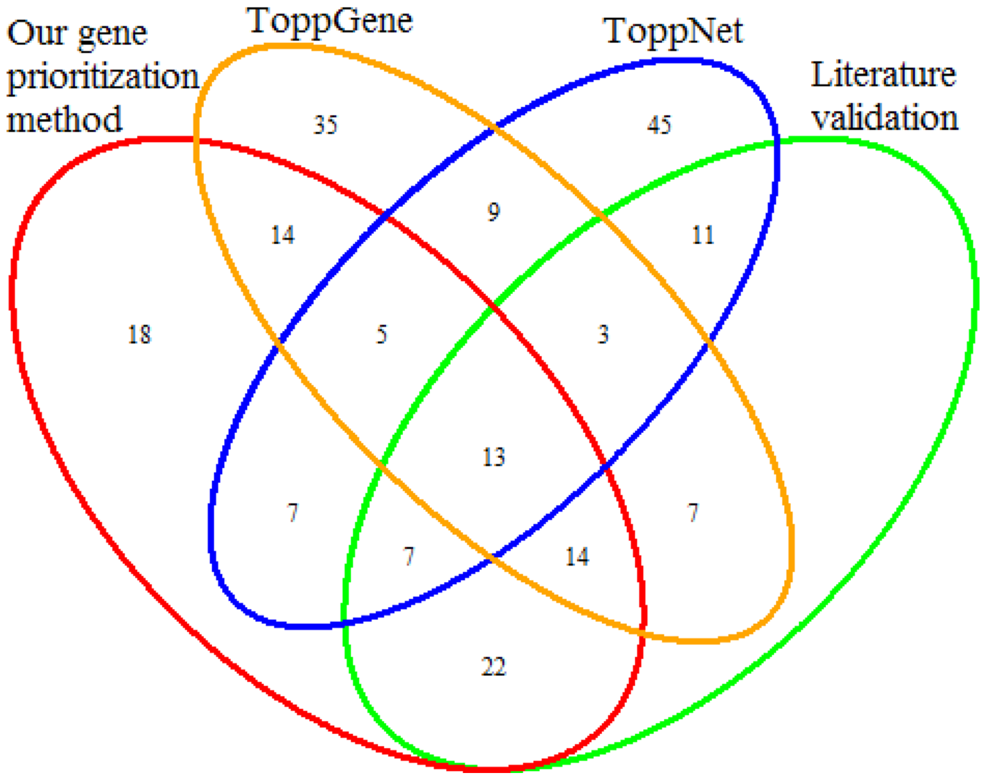

Supplementary Figure 1: Overlap of top 100 genes from our gene prioritization method, ToppGene and ToppNet, and their literature validation for the COPD-related metabolic network.

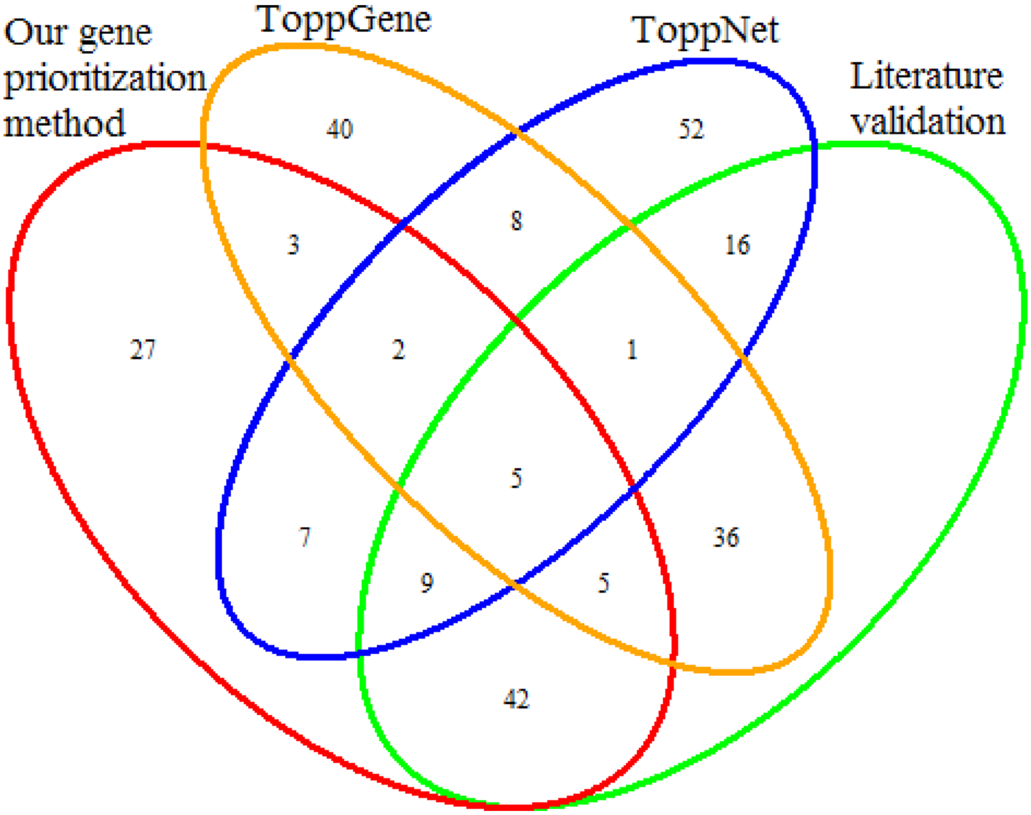

Supplementary Figure 2: Overlap of top 100 genes from our gene prioritization method, ToppGene and ToppNet, and their literature validation the COPD-related PPI network.

Supplementary Table 1: The number of significantly enriched functions and pathways, and the number and proportion of COPD-related functions and pathways in these significantly enriched functions and pathways, for top 100 genes from our gene prioritization method, ToppGene and ToppNet in COPD-related metabolic and PPI networks

		Function		Pathway	
		**Significantly enriched**	**COPD- related (proportion)**	**Significantly enriched**	**COPD- related (proportion)**
**COPD-related metabolic network**	**Our gene prioritization method**	143	86 (60.140%)	34	32 (94.118%)
	**ToppGene**	100	55 (55%)	20	15 (75%)
	**ToppNet**	75	42 (56%)	19	9 (47.368%)
**COPD-related PPI network**	**Our gene prioritization method**	541	223 (41.220%)	104	78 (75%)
	**ToppGene**	307	175 (57.003%)	53	42 (79.245%)
	**ToppNet**	134	76 (56.716%)	40	32 (80%)
